# The role of cystatin C in kidney injury in children and adolescents with type 1 diabetes mellitus: a systematic review

**DOI:** 10.1590/2175-8239-JBN-2024-0236en

**Published:** 2025-08-15

**Authors:** Nikolaos Gkiourtzis, Anastasia Stoimeni, Panagiota Michou, Maria Moutafi, Konstantinos Cheirakis, Aristeidis Christakopoulos, Agni Glava, Paraskevi Panagopoulou, Georgios Tsigaras, Assimina Galli-Tsinopoulou, Athanasios Christoforidis, Despoina Tramma

**Affiliations:** 1 Aristotle University of Thessaloniki, Faculty of Health Sciences, School of Medicine, 4th Department of Pediatrics, Papageorgiou General Hospital, Thessaloniki, Greece.; 2 Aristotle University of Thessaloniki, Faculty of Health Sciences, School of Medicine, Program of Postgraduate Studies Adolescent Medicine and Adolescent Health Care, Thessaloniki, Greece.; 3 Aristotle University of Thessaloniki, Faculty of Health Sciences, School of Medicine, 1st Department of Pediatrics, Hippokratio General Hospital, Thessaloniki, Greece.; 4 Aristotle University of Thessaloniki, Faculty of Health Sciences, School of Medicine, 2nd Department of Pediatrics, AHEPA University General Hospital, Thessaloniki, Greece.

**Keywords:** Cystatin C, Biomarkers, Diabetes Mellitus, Type 1, Diabetic Nephropathies, Pediatrics

## Abstract

**Introduction:**

Diabetic kidney disease (DKD) is a major complication of type 1 diabetes mellitus (T1D). In clinical practice, albuminuria and reduced estimated glomerular filtration rate (eGFR) are the main characteristics of DKD. Later studies revealed that interstitial damage is also observed as DKD occurs. Therefore, the application of a biomarker for early DKD detection was critical. This systematic review aimed to summarize the literature about the prognostic role of cystatin C in kidney injury in children and adolescents with T1D.

**Methods:**

From inception until September 24, 2024, an extensive literature search through major databases (MEDLINE/PubMed, Cochrane Library, and Scopus) was carried out to investigate the prognostic role of cystatin C in kidney injury in pediatric patients with T1D. The mean difference was used for continuous outcomes with 95%CI. A p < 0.05 was considered statistically significant. A quality assessment of included studies was conducted using the Newcastle-Ottawa Scale.

**Results:**

We included eleven studies with 2199 participants in this systematic review. The meta-analysis included four studies. No statistically significant difference was observed in serum cystatin C levels between patients with T1D and the control group.

**Conclusion:**

Although individual studies showed some benefit of using serum cystatin C for the prognosis of DKD in pediatric patients with T1D, the meta-analysis of included studies reached no statistical significance. Future clinical studies should focus on the prognostic role of cystatin C (serum and urinary) in identifying kidney injury in pediatric patients with T1D.

## INTRODUCTION

Diabetic kidney disease (DKD) is a major complication of diabetes mellitus (DM) with a high risk of end-stage kidney disease^1,2^. DKD occurs in 15–20% of pediatric patients with type 1 diabetes mellitus (T1D)^3^. In a recent cohort study, children with T1D had a 2.48 hazard ratio of early-onset kidney injury relative to children without DM^4^. In clinical practice, albuminuria and reduced estimated glomerular filtration rate (eGFR) are the main characteristics of DKD^2,5^. In early studies with kidney biopsy of patients with T1D, it was observed that glomerular and tubular basement membrane thickening occurred shortly after DM onset^6,7^. Later studies revealed that interstitial damage is also observed as DKD occurs^8^. Therefore, the application of a kidney biomarker for early DKD detection was critical. At first, animal experiments showed that cystatin C could detect early tubular injury in the setting of DKD9. Following studies presented the predictive and diagnostic value of cystatin C in kidney damage in type 2 DM (T2D) but none of them included pediatric patients^10,11^. This is the first systematic review examining the role of cystatin C in kidney injury in children and adolescents with T1D.

## METHODS

### Study Registration and Search Methodology

The study followed the Preferred Reporting Items for Systematic Reviews and Meta-Analyses (PRISMA) guidelines^12^. A prespecified protocol has been registered in OSF (https://osf.io/qes28/). Our search strategy included MEDLINE/PubMed, Cochrane Library, and Scopus databases from inception to September 24, 2024, using the following terms: (cystatin C) AND (type 1 diabetes mellitus). We screened all the references of the included studies as well as the related meta-analyses and systematic reviews for additional studies. We conducted an extensive search in Clinicaltrials.gov, PROSPERO, OSF, and “grey literature” to identify relevant studies and trials. Only studies published in English were included in the review.

### Eligibility Criteria

The research question (PICO) was set according to our prespecified protocol^13^. Clinical trials examining the role of cystatin C in kidney injury in pediatric patients and adolescents with T1D were included. Following our protocol, studies including adults or patients with T2D, case reports and series, systematic reviews, and meta-analyses were excluded.

### Study Procedure (Collection and Extraction of Data)

**Figure 1:**
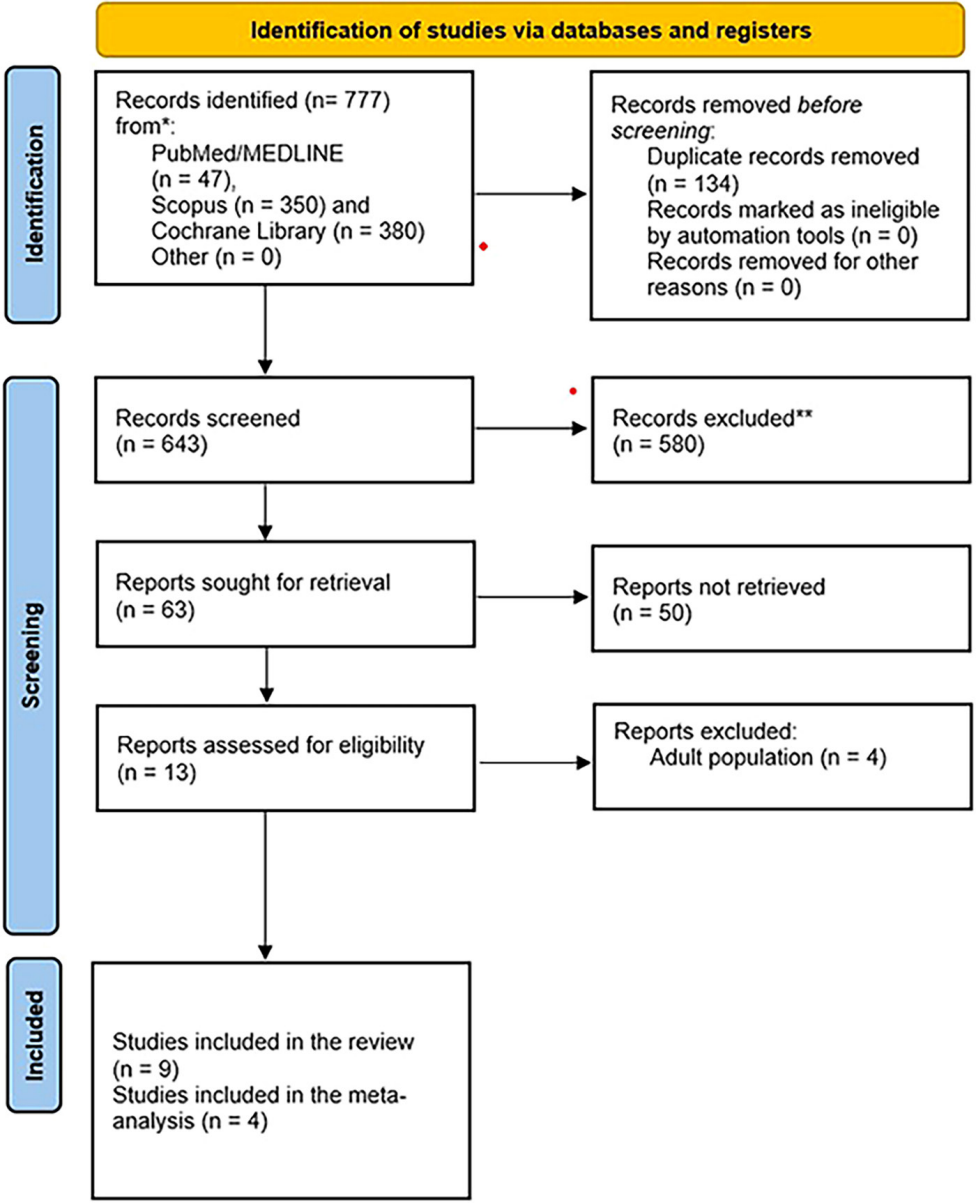
PRISMA 2020 flow chart.

Three independent reviewers (NG, AS, and MM) performed the literature search. Then, they extracted and imported all records into rayan.qcri.org, removing the duplicates^14^. The remaining studies were assessed initially by the title and abstract and then by full-text reading (Figure 1). If any disagreement arose, a fourth reviewer made the final decision. Finally, two independently working reviewers (PM, and AG) extracted the baseline characteristics of the eligible studies with the help of a pre-specified data extraction form. If any disagreements arose, they were solved with the help of a third reviewer. In case of missing data relevant to study characteristics, we contacted the corresponding authors to obtain them.

### Quality Assessment of Included Studies

The modified Newcastle-Ottawa Scale (NOS) was used for quality assessment of included studies. Three aspects are assessed with NOS: selection of the population, comparability between groups, and measurement of outcomes^15^. The maximum total score on this scale is 9 points (0–3 points for low quality, 4–6 points for medium quality, and 7–9 points for high quality).

### Outcome Measurements

The primary outcome was the difference in cystatin C (serum and urinary) levels between T1D patients and healthy controls. The secondary outcomes, determined a priori, included the comparison of urine protein to creatinine ratio (PCR), body mass index (BMI), HbA1C levels, duration of T1D, systolic and diastolic blood pressure (SBP and DBP) between the two groups and type of applied insulin therapy in patients with T1D.

### Statistical Analysis and Data Synthesis

A descriptive table is provided to summarize the baseline characteristics of the eligible studies. A meta-analysis was performed in R software (version 4.3.2 or later) using the “meta” package^16^. The relative prognostic role of cystatin C was estimated with mean difference (MD) and 95% confidence intervals (CI) for continuous outcomes. Forest plots illustrate the weighted outcomes with the 95%CI. Small study effects with publication bias were assessed visually with funnel plots and formally tested with Egger’s test^17^. Heterogeneity between the studies in pairwise meta-analysis was assessed using the I^2^ test (<40%: low, 30–60%: moderate, 50–90%: substantial, and 75–100%: considerable)^18^. A p < 0.05 was set as statistically significant.

## RESULTS

### Search Strategy Results

The extensive database search identified 1074 articles (Figure 1). After title and abstract screening, we assessed the full text of 18 studies for eligibility (Table S1). Finally, 11 studies published between 2011 and 2024 with 2199 participants were included in the systematic review^19–29^.

### Study Characteristics

Of the 11 studies, 6 were conducted in Europe^20,23,24,25,28,29^, two in the USA^21,26^, one in Egypt^27^, one in South Korea^19^, and one in multiple centers^22^. Participants’ mean age ranged from 10.5 (3.3) to 15.7 (4.0) years. Table 1 presents the characteristics of the included studies. Five studies directly compared patients with T1D^20,21,24,25,27^ and a healthy control group and four studies were included in the meta-analysis^20,21,25,27^.

**Table 1:** Study Characteristics

Study ID	Registration number	Country	Type of study	Mean age (SD)	Male (%)	Patients included in the study (N)	Study groups	BMI (kg/m^2^)	HbA1C (%)	DKA (yes/no)	Diabetes duration (years)	Presence of albuminuria (yes/no)	Determination of albuminuria (ACR or PCR)	GFR	Cystatin C (serum or urine)	Determination of biomarker
Chae et al.^19^	IRB number 4-2012-0001	South Korea	Cohort	15.7 (4.0)	41.8	98	T1D and T2D	N/A	8.4 (2.7)	No	7.1 (5.1)	Yes	ACR	(creatinine) 80.3 (32.9)	Serum Cystatin C	nephelometric immunoassay
Franchini et al.^20^	N/A	Italy	Case-control	T1D: 12.5 (3.1)	T1D: 45.7	235	T1D, Obese and Non-obese control	T1D: 21.2 (3.6)	T1D: 7.7 (8.4)	No	N/A	Yes	N/A	(creatinine) T1D: 113.4 (19.1)	Serum Cystatin C	nephelometric immunoassay
				Control: 12.6 (3.0)	Control: 40.8			Control: 21.4 (4.2)	Control: 4.12 (0.1)					(creatinine) Control: 104.7 (18.9)		
Maahs et al.^21^	N/A	USA	Case-control	T1D: 15.4 (2.2)	T1D: 12.5 (3.1)	337	T1D and Non-diabetic control	N/A	T1D: 8.9 (1.6)	No	8.7 (2.9)	Yes	ACR	N/A	Serum Cystatin C	nephelometric immunoassay
				Control: 15.5 (2.2)	Control: 12.6 (3.0)				Control: 5.3 (0.3)							
Marcovecchio et al.^22^	N/A	UK, Australia, Canada	Cohort	Upper tertile: 13.9 (1.6)	Upper tertile: 52.5	629	T1D (Upper tertile and Lower-middle tertile)	Upper tertile: 21.4 (3.7)	Upper tertile: 13.9 (1.6)	No	Upper tertile: 5.9 (3.1)	Yes	ACR	(eGFR) Upper tertile: 137.0 (23.9)	Serum Cystatin C	nephelometric immunoassay
				Lower-middle tertile: 14.3 (1.7)	Lower-middle tertile: 53.2			Lower-middle tertile: 22.0 (3.9)	Lower-middle tertile: 14.3 (1.7)		Lower-middle tertile: 7.5 (3.4)			(eGFR) Lower-middle tertile: 129.3 (22.4)		
Nilsson and Dereke^23^	2006/599, 2013/693 and 2014/822	Sweden	Cohort	<5 years: 11.0 (4.5)	<5 years: 53.3	244	T1D (<5, 5-10 and >10 years of T1D duration)	<5 years: 19.1 (4.6)	<5 years: 8.4 (2.5)	No	<5 years: 1 (2.2)	N/A	N/A	N/A	Serum Cystatin C	ELISA
				5-10 years: 13.5 (2.8)	5-10 years: 63.0			5-10 years: 21.0 (4.3)	5-10 years: 7.3 (1.0)		5-10 years: 7 (1.5)					
				>10 years: 15.2 (2.3)	>10 years: 52.3			>10 years: 22.4 (4.2)	>10 years: 7.7 (0.8)		>10 years: 11.3 (2.3)					
Papadopoulou-Marketou et al.^24^	Ethics Committee of the Aghia Sophia Children’s Hospital	Greece	Observational follow-up	T1D: 13.1 (3.2)	T1D: 57.1	105	T1D and Healthy controls	N/A	T1D: 8.36 (1.7)	No	4.59 (3.49)	Yes	ACR	(eGFR)T1D: 90.72 (19.8)	Serum Cystatin C	ELISA, nephelometric immunoassay
				Control: 12.8 (6,6)	Control: 42.8				Control: 4.7 (0.4)					Control: N/A		
Papadopoulou-Marketou et al.^25^	Ethics Committee of the Aghia Sophia Children’s Hospital	Greece	Cross-sectional prospective long-term follow-up	T1D: 13.9 (1.9)	T1D: 56.14	106	T1D and Healthy control	N/A	T1D: 9.1 (1.8)	No	5.4 (3.3)	Yes	ACR	(eGFR) T1D: 110.1 (14.6)	Serum Cystatin C	ELISA, nephelometric immunoassay
				Control: 10.5 (3.3)	Control: 57.14				Control: 4.65					(eGFR) Control: 103.5 (14.5)		
Piani et al.^26^	N/A	USA	Cross-sectional	11.0 (4.0)	52.5	40	DKA patients	18.7 (4.5)	12.7 (1.9)	Yes	N/A	No	N/A	(eGFR) 139.5 (5.7)	Serum Cystatin C	Sandwich immunoassay
Salem et al.^27^	MS/15.12.52	Egypt	Case-control	Normoalbuminuric: 12.9 (1.9)	Normoalbuminuric: 62.1	90	T1D (normoalbuminuric and microalbuminuric) and Control	N/A	Normoalbuminuric: 7.4 (0.6)	No	N/A	Yes	ACR	(creatinine) Normoalbuminuric: 103.1 (33.3)	Serum Cystatin C	ELISA
				Microalbuminuric: 13.4 (1.5)	Microalbuminuric: 54.8				Microalbuminuric: 7.9 (0.6)					(creatinine) Microalbuminuric: 100.7 (27.9)		
														(creatinine) Control: 113.9 (30.2)		
				Control: 13.8 (2.4)	Control: 36.7				Control: 4.9 (0.3)							
Słomin´ski et al.^28^	N/A	Poland	Cohort	Nephropathy-free: 12.7 (2.7)	50	240	Nephropathy-free and Nephropathy	Nephropathy-free: 18.9 (2.4)	Nephropathy-free: 8.4 (1.6)	No	Nephropathy-free: 7.0 (2.8)	Yes	N/A	(eGFR) Nephropathy-free: 121 (38)	Serum Cystatin C	ELISA
				Nephropathy: 13.7 (3.6)				Nephropathy: 19.7 (2.8)	Nephropathy: 8.3 (1.2)		Nephropathy: 8.8 (3.0)			(eGFR) Nephropathy: 110 (28)		
Trutin et al.^29^	N/A	Croatia	Cross-sectional	13.6 (3.8)	39	75	T1D	N/A	7.6 (1.2)	No	6.3 (3.8)	Yes	N/A	(eGFR) 110 (19.2)	Serum Cystatin C	PETIA

Abbreviations – ACR: Albumin to Creatinine Ratio; BMI: body mass index; DKA: Diabetic Ketoacidosis; ELISA: Enzyme-Linked Immunosorbent Assay; GFR: Glomerular Filtration Rate; HbA1C: Hemoglobin A1C; ID: identification; N/A: Not Applicable; PETIA: Particle-enhanced Turbidimetric Immunoassay; PCR: Protein to Creatinine Ratio; SD: standard difference; T1D: Type 1 Diabetes; T2D: Type 2 Diabetes; UK: United Kingdom; USA: United States of America.

Note – GFR calculated with creatinine, cystatin C, or inulin or eGFR calculated with creatinine) (mL/min/1.73 m^2^).

### Quality Assessment of the Included Studies

The quality assessment of the 11 included studies using the NOS scale is presented in Table 2. Only four studies were categorized as high quality^19,22,23,28^, five studies as medium quality^20,21,24,25,27^, and two were considered to have low quality^26,29^.

**Table 2:** Quality assessment of included studies

First author & year	Is the case definition adequate?	Representativeness of the cases	Selection of controls	Definition of controls	Comparability of cases and controls based on the design or analysis	Ascertainment of exposure	Same method of ascertainment for cases and controls	Non-response rate	Total score
Chae 2012^19^	*	*	*	*	*	*	*		7/9
Franchini 2015^20^	*	*		*	*	*	*		6/9
Maahs 2011^21^	*	*			*	*	*		5/9
Marcovecchio 2014^22^	*	*	*	*	*	*	*		7/9
Nilsson 2024^23^	*	*	*	*	*	*	*		7/9
Papadopoulou-Marketou 2014^24^	*	*		*	*	*	*		6/9
Papadopoulou-Marketou 2017^25^	*	*		*	*	*	*		6/9
Piani 2021^26^	*	*				*			3/9
Salem 2020^27^	*	*		*	*	*	*		6/9
Słomin´ski 2018^28^	*	*	*	*	*	*	*		7/9
Trutin 2022^29^	*	*				*			3/9

### Analysis of the Primary Outcome

**Figure 2:**
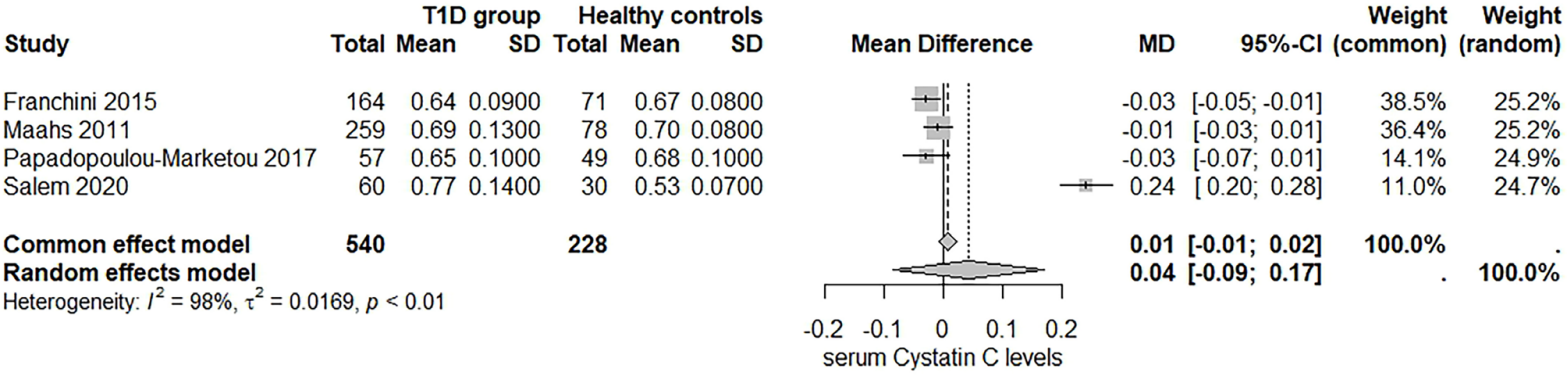
Forest plot assessing the prognostic role of serum cystatin C in diabetic kidney disease (DKD).

A total of four studies evaluated the association between serum cystatin C levels in individuals with T1D and healthy controls. The pooled estimate indicated that the mean serum cystatin C level in the T1D group was 0.04 mg/L higher than in the control group. However, this difference was not statistically significant (Figure 2). Additionally, there was substantial heterogeneity among the studies (I^2^ = 98%), suggesting a significant variability in the results. An analysis of urinary cystatin C was not possible as it was not examined in any of the included studies.

**Figure 3:**
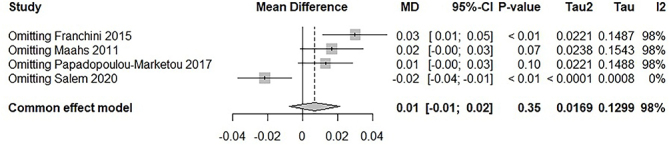
Forest plot assessing a sensitivity analysis of the primary outcome.

**Figure 4:**
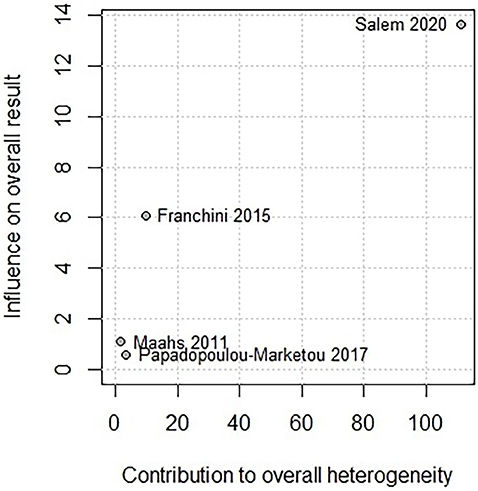
Baujat plot of the heterogeneity in primary outcome.

A sensitivity analysis (Figure 3) revealed that omitting the study by Salem et al.^27^ substantially reduced the heterogeneity. Further exploration using the Baujat plot (Figure 4) confirmed that Salem et al.^27^ had a notable impact on both the overall result and the heterogeneity. Despite its significant influence, the study was not excluded from the meta-analysis due to its importance in the overall effect estimation. Publication bias assessment was not statistically significant (p = 0.1526).

### Analysis of Secondary Outcomes

**Figure 5:**
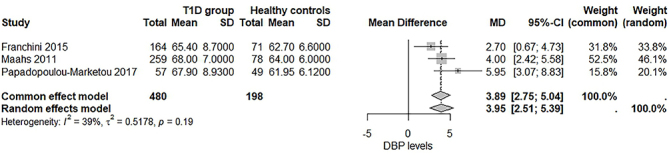
Forest plot assessing diastolic blood pressure (DBP) levels in type 1 diabetes mellitus (T1D) patients versus healthy controls.

Patients with T1D exhibited DBP levels that were 3.95 mmHg higher than healthy controls, and this difference was statistically significant (95%CI = [2.51–5.39], I^2^ = 39%) (Figure 5). In contrast, the mean difference in SBP levels between the two groups did not reach statistical significance (Figure S1). The urine PCR, BMI, and HbA1C levels, the duration of T1D, and type of applied insulin therapy in T1D patients could not be examined as there were no sufficient data.

## DISCUSSION

Nephropathy caused by T1D, also known as DKD, is a well-described and common complication. Pediatric patients with T1D should be evaluated at 11 years of age with 2–5 years of T1D duration or at puberty^30^. The earliest manifestation of DKD is albuminuria, but some patients may present a particular type of normoproteinuric DKD [urine albumin to creatinine ratio (ACR) ≤30 mg/g and eGFR <60 mL/min/1.73 m^2^]^31,32^. DKD is generally detectable after the development of microalbuminuria or by decreased GFR in patients with T1D^3,33^. However, urine microalbumin may be affected by urine retention, high blood pressure, and urinary tract infections^34^. In addition, GFR is mainly estimated by serum creatinine, which may be affected by muscle mass, gender, age, and medication^35^. Creatinine-calculated eGFR is often considered to be closely correlated with the true GFR. However, it has inaccuracies, especially in children, due to ethnicity, muscle mass, age, gender, and dietary differences^36^. Cystatin C serves as an alternative endogenous marker that remains independent of muscle mass, physical activity, and protein intake, exhibiting minimal influence from age and gender^36,37^. Taking into consideration the hormonal and growth alterations during adolescence, we hypothesize that cystatin C may be a better predictor of CKD progression than GFR or eGFR in this age group. Therefore, the application of novel DKD markers in clinical practice was necessary. Liao et al.^35^ meta-analysis revealed that cystatin C has a great diagnostic value for DKD in adults with T1D and T2D, as only two of the included studies examined the prognostic role of cystatin C in pediatric patients with T1D^19,27^.

Our systematic review included all the available studies examining the role of cystatin C in pediatric patients with T1D. No other study, consensus, or expert opinion paper has reviewed or analyzed this topic including all the available studies of patients with T1D under 21 years of age according to the Bright Futures guidelines from the American Academy of Pediatrics^38^.

Only five of the 11 studies in the systematic review directly compared patients with T1D and a healthy control group^20,21,24,25,27^. The rest of the studies compared patients with T1D either with patients with T2D^19^ or with other patients with T1D (with or without albuminuria^22^, according to the duration of T1D^23^, with acute kidney injury during diabetic ketoacidosis^26^, with or without DKD^28^, and risk for DKD^29^).

Five studies in this review found that serum cystatin C had a positive correlation or statistical significance with the prognosis or diagnosis of kidney impairment in pediatric patients with T1D^19,23,24,27,29^. Specifically, Chae et al.^19^ showed that serum cystatin C was more elevated in patients with DM and microalbuminuria (p = 0.04), although there was no separation of patients with T1D or T2D in this comparison. In Nilsson and Dereke’s study^23^, cystatin C levels were elevated in patients with T1D with diabetes duration >5 years (p < 0.001) among pediatric patients with T1D. In the Papadopoulou-Marketou et al.^24^ study, serum cystatin C was positively correlated with NGAL (p < 0.001) and creatinine (p = 0.009) but negatively correlated with eGFR (p = 0.025) in patients with T1D compared with healthy individuals. Furthermore, in the Salem et al.^27^ study, serum cystatin C (p < 0.001) and eGFR-cystatin C (p < 0.001) were significantly higher in children with microalbuminuria than in the normoalbuminuric children with T1D and healthy controls. In children and adolescents with T1D in Trutin et al.^29^, cystatin C was significantly associated with the estimated risk of DKD (p = 0.009). In contrast, Franchini et al.^20^ showed that patients with T1D had lower levels of serum cystatin C than the control group (p < 0.001), although after adjusting for fasting glycemia, the difference between the groups lost significance. Additionally, in the Maahs et al.^21^ and Papadopoulou-Marketou et al.^25^ studies, serum cystatin C levels were similar in patients with and without T1D.

Despite the significant results of individual studies regarding the benefit of serum cystatin C in detection or prognosis of DKD in pediatric patients with T1D, our systematic review has limitations that must be acknowledged. Only four studies were included in the meta-analysis^20,21,25,27^. Additionally, this meta-analysis showed no statistical significance of serum cystatin C in the prognosis of DKD in pediatric patients with T1D. Specifically, only Salem et al.^27^ showed that serum cystatin C may lead to early detection of DKD even before the development of albuminuria in childhood. A possible explanation is that the included studies used different approaches for cystatin C evaluation such as eGFR-cystatin C, NGAL-cystatin C, creatinine-cystatin C, and ROC curve analysis of serum cystatin C rather than serum cystatin C or eGFR calculated with cystatin C analysis. The quality assessment of the included study showed that only four of the 11 studies were categorized as high quality^19,22,23,28^. Unfortunately, no separation and further analysis of patients with or without hyperfiltration and patients with or without microalbuminuria could be made. Finally, none of the included studies mentioned the importance of urinary cystatin C as a tubular biomarker in DKD^10,11^ and most of the prespecified secondary outcomes were not examined.

## CONCLUSION

In conclusion, although individual studies showed some benefit of using serum cystatin C in the prognosis of DKD in pediatric patients with T1D, the meta-analysis of included studies reached no statistical significance. Future large clinical studies, examining the prognostic role of cystatin C (both serum and urinary) comparing children and adolescents with T1D and healthy controls should be conducted to confirm its beneficial effect on DKD prognosis.

## Data Availability

Data are available online or after request from the corresponding author.

## Supplementary Material

Figure S1 – Forest plot assessing systolic blood pressure (SBP) levels in type 1 diabetes mellitus (T1D) patients versus healthy controls.

Table S1 – Excluded studies and reason for exclusion.
