# Supplementary material for: The role of cystatin C in kidney injury in children and adolescents with type 1 diabetes mellitus: a systematic review
Source: J Bras Nefrol. 2025 Aug 15;47(4):e20240236. doi: 10.1590/2175-8239-JBN-2024-0236en (PMC12360829; doi:10.1590/2175-8239-JBN-2024-0236en)
Supplement: Supplementary file 1 [file 2175-8239-jbn-47-4-e20240236-suppl1.pdf]

Braz. J. Nephrol.
https://doi.org/10.1590/2175-8239-JBN-2024-0236en

Supplementary Material to "The role of cystatin C in kidney injury in children and adolescents with type 1 diabetes mellitus: a systematic review"

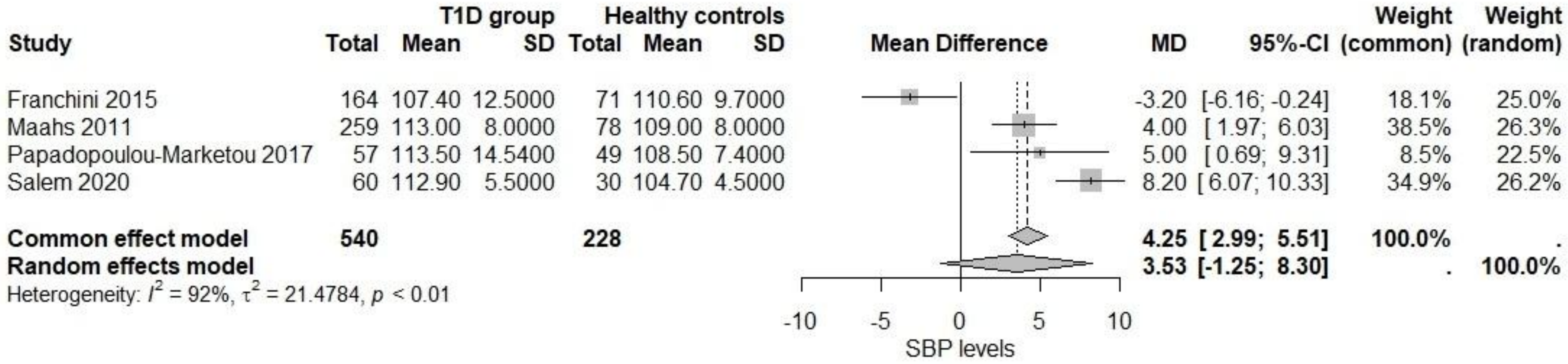

Figure S1 - Forest plot assessing the SBP levels in T1D patients versus healthy controls; SBP: systolic blood pressure; T1D: type 1 diabetes mellitus.
