# Supplementary material for: The role of cystatin C in kidney injury in children and adolescents with type 1 diabetes mellitus: a systematic review
Source: J Bras Nefrol. 2025 Aug 15;47(4):e20240236. doi: 10.1590/2175-8239-JBN-2024-0236en (PMC12360829; doi:10.1590/2175-8239-JBN-2024-0236en)
Supplement: Supplementary file 3 [file 2175-8239-jbn-47-4-e20240236-suppl3.pdf]

Braz. J. Nephrol.
https://doi.org/10.1590/2175-8239-JBN-2024-0236pt

Material Suplementar para "O papel da cistatina C na injúria renal em crianças e adolescentes com diabetes mellitus tipo 1: uma revisão sistemática"

Tabela S1 – Estudos excluídos e motivo da exclusão.

**Estudo**	**Motivo da exclusão**
Cheng, Y., Xu, C., Wang, S., Hou, L., Guan, Q., & Zhou, X. (2020). *Serum cystatin C levels are decreased in type 1 diabetes mellitus patients with diabetic ketoacidosis*. *Minerva endocrinologica*, *45*(2), 106–116. https://doi.org/10.23736/S0391-1977.20.03147-8	População adulta
Christensson, A. G., Grubb, A. O., Nilsson, J. A., Norrgren, K., Sterner, G., & Sundkvist, G. (2004). *Serum cystatin C advantageous compared with serum creatinine in the detection of mild but not severe diabetic nephropathy*. *Journal of internal medicine*, *256*(6), 510–518. https://doi.org/10.1111/j.1365-2796.2004.01414.x	População adulta
Marcovecchio, M. L., Colombo, M., Dalton, R. N., McKeigue, P. M., Benitez-Aguirre, P., Cameron, F. J., Chiesa, S. T., Couper, J. J., Craig, M. E., Daneman, D., Davis, E. A., Deanfield, J. E., Donaghue, K. C., Jones, T. W., Mahmud, F. H., Marshall, S. M., Neil, A., Colhoun, H. M., Dunger, D. B., & AdDIT and the SDRNT1BIO Investigators (2020). *Biomarkers associated with early stages of kidney disease in adolescents with type 1 diabetes*. *Pediatric diabetes*, *21*(7), 1322–1332. https://doi.org/10.1111/pedi.13095	População adulta
Rigalleau, V., Beauvieux, M. C., Lasseur, C., Chauveau, P., Raffaitin, C., Perlemoine, C., Barthe, N., Combe, C., & Gin, H. (2007). *The combination of cystatin C and serum creatinine improves the monitoring of kidney function in patients with diabetes and chronic kidney disease*. *Clinical chemistry*, *53*(11), 1988–1989. https://doi.org/10.1373/clinchem.2007.092171	População adulta
Stankute, I., Radzeviciene, L., Monstaviciene, A., Dobrovolskiene, R., Danyte, E., & Verkauskiene, R. (2022). *Serum Cystatin C as a Biomarker for Early Diabetic Kidney Disease and Dyslipidemia in Young Type 1 Diabetes Patients*. *Medicina (Kaunas, Lithuania)*, *58*(2), 218. https://doi.org/10.3390/medicina58020218	População adulta
