# Supplementary material for: The role of cystatin C in kidney injury in children and adolescents with type 1 diabetes mellitus: a systematic review
Source: J Bras Nefrol. 2025 Aug 15;47(4):e20240236. doi: 10.1590/2175-8239-JBN-2024-0236en (PMC12360829; doi:10.1590/2175-8239-JBN-2024-0236en)
Supplement: Supplementary file 4 [file 2175-8239-jbn-47-4-e20240236-suppl4.pdf]

Braz. J. Nephrol.
https://doi.org/10.1590/2175-8239-JBN-2024-0236pt

Material Suplementar para "O papel da cistatina C na injúria renal em crianças e adolescentes com diabetes mellitus tipo 1: uma revisão sistemática"

	Grupo com DM1			Grupo controle						
**Estudo**	**Total**	**Média**	**DP**	**Total**	**Média**	**DP**	**DM**	**IC 95%**	**Peso (fixo)**	**Peso (aleatório)**
Franchini 2015	164	107,40	12,5000	71	110,60	9,7000	-3,20	[-6,16; -0,24]	18,1%	25,0%
Maahs 2011	259	113,00	8,0000	78	109,00	8,0000	4,00	[1,97; 6,03]	38,5%	26,3%
Papadopoulou-Marketou 2017	57	113,50	14,5400	49	108,50	7,4000	5,00	[0,69; 9,31]	8,5%	22,5%
Salem 2020	60	112,90	5,5000	30	104,70	4,5000	8,20	[6,07; 10,33]	34,9%	26,2%
**Modelo de efeitos fixos**	**540**			**228**			**4,25**	**[2,99; 5,51]**	**100,0%**	**.**
**Modelo de efeitos aleatórios**							**3,53**	**[-1,25; 8,30]**	**.**	**100,0%**

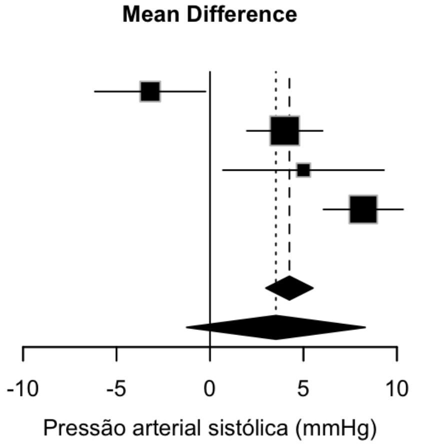

Figura S1 - Gráfico de floresta avaliando os níveis de PAS em pacientes com DM1 versus controles saudáveis; PAS: pressão arterial sistólica; DM1: diabetes mellitus tipo 1.
