# Supplementary material for: The role of cystatin C in kidney injury in children and adolescents with type 1 diabetes mellitus: a systematic review
Source: J Bras Nefrol. 2025 Aug 15;47(4):e20240236. doi: 10.1590/2175-8239-JBN-2024-0236en (PMC12360829; doi:10.1590/2175-8239-JBN-2024-0236en)
Supplement: Translation

# Translation: O papel da cistatina C na injúria renal em crianças e adolescentes com diabetes mellitus tipo 1: uma revisão sistemática

**DOI:** 10.1590/2175-8239-JBN-2024-0236pt

**Authors:** Nikolaos Gkiourtzis, Anastasia Stoimeni, Panagiota Michou, Maria Moutafi, Konstantinos Cheirakis, Aristeidis Christakopoulos, Agni Glava, Paraskevi Panagopoulou, Georgios Tsigaras, Assimina Galli-Tsinopoulou, Athanasios Christoforidis, Despoina Tramma

**Affiliations:** 1 Aristotle University of Thessaloniki, Faculty of Health Sciences, School of Medicine, 4th Department of Pediatrics, Papageorgiou General Hospital, Thessaloniki, Greece.; 2 Aristotle University of Thessaloniki, Faculty of Health Sciences, School of Medicine, Program of Postgraduate Studies Adolescent Medicine and Adolescent Health Care, Thessaloniki, Greece.; 3 Aristotle University of Thessaloniki, Faculty of Health Sciences, School of Medicine, 1st Department of Pediatrics, Hippokratio General Hospital, Thessaloniki, Greece.; 4 Aristotle University of Thessaloniki, Faculty of Health Sciences, School of Medicine, 2nd Department of Pediatrics, AHEPA University General Hospital, Thessaloniki, Greece.

**Keywords:** Cistatina C, Biomarcadores, Diabetes Mellitus Tipo 1, Nefropatias Diabéticas, Pediatria

## Abstract

**Introdução:**

Doença renal diabética (DRD) é uma das principais complicações do diabetes mellitus tipo 1 (DM1). Na prática clínica, albuminúria e taxa de filtração glomerular estimada (TFGe) reduzida são as principais características da DRD. Estudos posteriores revelaram que a lesão intersticial também é observada quando ocorre DRD. Portanto, a aplicação de um biomarcador para a detecção precoce da DRD foi fundamental. Esta revisão sistemática teve como objetivo resumir a literatura sobre o papel prognóstico da cistatina C na injúria renal em crianças e adolescentes com DM1.

**Métodos:**

Desde o início até 24 de setembro de 2024, realizou-se extensa pesquisa bibliográfica nos principais bancos de dados (MEDLINE/PubMed, *Cochrane Library* e *Scopus*) para investigar o papel prognóstico da cistatina C na injúria renal em pacientes pediátricos com DM1. A diferença média foi utilizada para desfechos contínuos com IC95%. Um valor p < 0,05 foi considerado estatisticamente significativo. A avaliação da qualidade dos estudos incluídos foi realizada por meio da Escala de Newcastle-Ottawa.

**Resultados:**

Incluímos onze estudos com 2.199 participantes nessa revisão sistemática. A meta-análise incluiu quatro estudos. Não houve diferença estatisticamente significativa nos níveis séricos de cistatina C entre os pacientes com DM1 e o grupo controle.

**Conclusão:**

Embora estudos individuais tenham demonstrado algum benefício do uso da cistatina C sérica para o prognóstico de DRD em pacientes pediátricos com DM1, a meta-análise dos estudos incluídos não alcançou significância estatística. Estudos clínicos futuros devem concentrar-se no papel prognóstico da cistatina C (sérica e urinária) na identificação de injúria renal em pacientes pediátricos com DM1.

## Introdução

A doença renal diabética (DRD) é uma das principais complicações do diabetes mellitus (DM), com alto risco de doença renal em estágio terminal^1,2^. A DRD ocorre em 15–20% dos pacientes pediátricos com diabetes mellitus tipo 1 (DM1)^3^. Em um estudo de coorte recente, crianças com DM1 apresentaram uma razão de risco de 2,48 para injúria renal de início precoce em relação a crianças sem DM^4^. Na prática clínica, a albuminúria e a redução da taxa de filtração glomerular estimada (TFGe) são as principais características da DRD^2,5^. Em estudos iniciais com biópsias renais de pacientes com DM1, observou-se que o espessamento das membranas basais glomerular e tubular ocorreu logo após o início do DM^6,7^. Estudos posteriores revelaram que a lesão intersticial também é observada à medida que a DRD ocorre^8^. Portanto, a aplicação de um biomarcador renal para a detecção precoce da DRD foi fundamental. Inicialmente, experimentos em modelos animais demonstraram que a cistatina C pode detectar lesões tubulares precoces no contexto da DRD^9^. Os estudos subsequentes apresentaram o valor preditivo e diagnóstico da cistatina C na lesão renal no DM tipo 2 (DM2), mas nenhum deles incluiu pacientes pediátricos^10,11^. Esta é a primeira revisão sistemática a examinar o papel da cistatina C na injúria renal em crianças e adolescentes com DM1.

## Métodos

### Registro do Estudo e Metodologia de Pesquisa

O estudo seguiu as diretrizes do *Preferred Reporting Items for Systematic Reviews and Meta-Analyses* (PRISMA)^12^. Um protocolo predefinido foi registrado na plataforma OSF (https://osf.io/qes28/). Nossa estratégia de busca incluiu os bancos de dados MEDLINE/PubMed, *Cochrane Library* e *Scopus* desde o início até 24 de setembro de 2024, utilizando os seguintes termos: (cistatina C) E (diabetes mellitus tipo 1). Foram examinadas todas as referências dos estudos incluídos, bem como as meta-análises e revisões sistemáticas relacionadas, em busca de estudos adicionais. Realizamos uma extensa pesquisa no Clinicaltrials.gov, PROSPERO, OSF e na “literatura cinzenta” para identificar estudos e ensaios relevantes. Somente estudos publicados em inglês foram incluídos na revisão.

### Critérios de Elegibilidade

A pergunta de pesquisa (PICO) foi definida de acordo com nosso protocolo predefinido^13^. Foram incluídos ensaios clínicos que examinaram o papel da cistatina C na injúria renal em pacientes pediátricos e adolescentes com DM1. Seguindo nosso protocolo, foram excluídos estudos que incluíam adultos ou pacientes com DM2, relatos e séries de casos, revisões sistemáticas e meta-análises.

### Procedimento do Estudo (Coleta e Extração de Dados)

Três revisores independentes (NG, AS e MM) realizaram a pesquisa na literatura. Em seguida, todos os registros foram extraídos e importados para a plataforma rayan.qcri.org, sendo removidas as duplicatas^14^. Os demais estudos foram avaliados inicialmente pelo título e resumo e, posteriormente, pela leitura do texto completo (Figura 1). Em caso de discordância, um quarto revisor foi responsável pela decisão final. Por fim, dois revisores independentes (PM e AG) extraíram as características basais dos estudos elegíveis com o auxílio de um formulário de extração de dados predefinido. Eventuais divergências foram resolvidas com a ajuda de um terceiro revisor. No caso de dados ausentes que fossem relevantes para as características do estudo, entramos em contato com os autores correspondentes para obtê-los.

**Figura 1:** Fluxograma PRISMA 2020.

### Avaliação da Qualidade dos Estudos Incluídos

A Escala de Newcastle-Ottawa (NOS) modificada foi utilizada para a avaliação da qualidade dos estudos incluídos. Três aspectos são avaliados com a NOS: seleção da população, comparabilidade entre os grupos e mensuração dos desfechos^15^. A pontuação total máxima dessa escala é de 9 pontos (0-3 pontos indica baixa qualidade, 4-6 pontos, qualidade moderada, e 7-9 pontos, alta qualidade).

### Medidas de Desfechos

O desfecho primário foi a diferença nos níveis de cistatina C (sérica e urinária) entre pacientes com DM1 e controles saudáveis. Os desfechos secundários, determinados a priori, incluíram a comparação entre os dois grupos da relação proteína/creatinina (RPC) urinária, índice de massa corporal (IMC), níveis de HbA1C, duração do DM1, pressão arterial sistólica e diastólica (PAS e PAD), além do tipo de insulinoterapia aplicada em pacientes com DM1.

### Análise Estatística e Síntese dos Dados

Uma tabela descritiva é apresentada para resumir as características basais dos estudos elegíveis. Foi realizada uma meta-análise no software R (versão 4.3.2 ou posterior) utilizando o pacote “meta”^16^. O papel prognóstico relativo da cistatina C foi estimado com diferença média (MD) e intervalos de confiança (IC) de 95% para desfechos contínuos. Os gráficos de floresta ilustram os desfechos ponderados com o IC de 95%. Os efeitos de estudos de pequeno porte com viés de publicação foram avaliados visualmente por meio de gráficos de funil e testados formalmente com o teste de Egger^17^. A heterogeneidade entre os estudos na meta-análise pareada foi avaliada usando o teste I^2^ (<40%: baixa, 30–60%: moderada, 50–90%: substancial e 75–100%: considerável)^18^. Um valor de p < 0,05 foi definido como estatisticamente significativo.

## RESULTADOS

### Resultados da Estratégia de Busca

A extensa pesquisa no banco de dados identificou 1.074 artigos (Figura 1). Após a triagem dos títulos e resumos, o texto completo de 18 estudos foi avaliado quanto à elegibilidade (Tabela S1). Por fim, 11 estudos publicados entre 2011 e 2024, com um total de 2.199 participantes, foram incluídos na revisão sistemática^19,20,21,22,23,24,25,26,27,28, 29^.

### Características do Estudo

Dos 11 estudos, 6 foram realizados na Europa^20,23,24,25,28,29^, dois nos EUA^21,26^, um no Egito^27^, um na Coreia do Sul^19^ e um em múltiplos centros^22^. A idade média dos participantes variou de 10,5 (3,3) a 15,7 (4,0) anos. A Tabela 1 apresenta as características dos estudos incluídos. Cinco estudos compararam diretamente pacientes com DM1^20,21,24,25,27^ e um grupo controle saudável, e quatro estudos foram incluídos na meta-análise^20,21,25,27^.

**Tabela 1:** Características do estudo

ID do estudo	Número de registro	País	Tipo de estudo	Idade média (DP)	Homens (%)	Pacientes incluídos no estudo (N)	Grupos de estudo	IMC (kg/m^2^)	HbA1C (%)	CAD (sim/não)	Duração do diabetes (anos)	Presença de albuminúria (sim/não)	Determinação da albuminúria (RAC ou RPC)	TFG	Cistatina C (sérica ou urinária)	Determinação do biomarcador
Chae et al.^19^	Número IRB 4-2012-0001	Coreia do Sul	Coorte	15,7 (4,0)	41,8	98	DM1 e DM2	N/A	8,4 (2,7)	Não	7,1 (5,1)	Sim	RAC	(creatinina) 80,3 (32,9)	Cistatina C sérica	imunoensaio nefelométrico
Franchini et al.^20^	N/A	Itália	Caso-controle	DM1: 12,5 (3,1)	DM1: 45,7	235	DM1, Controle Obeso e Não Obeso	DM1: 21,2 (3,6)	DM1: 7,7 (8,4)	Não	N/A	Sim	N/A	(creatinina) DM1: 113,4 (19,1)	Cistatina C sérica	imunoensaio nefelométrico
				Controle: 12,6 (3,0)	Controle: 40,8			Controle: 21,4 (4,2)	Controle: 4,12 (0,1)					(creatinina) Controle: 104,7 (18,9)		
Maahs et al.^21^	N/A	EUA	Caso-controle	DM1: 15,4 (2,2)	DM1: 12,5 (3,1)	337	DM1 e Controle não diabético	N/A	DM1: 8,9 (1,6)	Não	8,7 (2,9)	Sim	RAC	N/A	Cistatina C sérica	imunoensaio nefelométrico
				Controle: 15,5 (2,2)	Controle: 12,6 (3,0)				Controle: 5,3 (0,3)							
Marcovecchio et al.^22^	N/A	UK, Austrália, Canadá	Coorte	Tercil superior: 13,9 (1,6)	Tercil superior: 52,5	629	DM1 (tercil superior e tercil médio-inferior)	Tercil superior: 21,4 (3,7)	Tercil superior: 13,9 (1,6)	Não	Tercil superior: 5,9 (3,1)	Sim	RAC	(TFGe) Tercil superior: 137,0 (23,9)	Cistatina C sérica	imunoensaio nefelométrico
				Tercil médio-baixo: 14,3 (1,7)	Tercil médio-baixo: 53,2			Tercil médio-baixo: 22,0 (3,9)	Tercil médio-baixo: 14,3 (1,7)		Tercil médio-baixo: 7,5 (3,4)			(TFGe) Tercil médio-baixo: 129,3 (22,4)		
Nilsson e Dereke^23^	2006/599, 2013/693 e 2014/822	Suécia	Coorte	<5 anos: 11,0 (4,5)	<5 anos: 53,3	244	DM1 (<5, 5-10 e >10 anos de duração do DM1)	<5 anos: 19,1 (4,6)	<5 anos: 8,4 (2,5)	Não	<5 anos: 1 (2,2)	N/A	N/A	N/A	Cistatina C sérica	ELISA
				5-10 anos: 13,5 (2,8)	5-10 anos: 63,0			5-10 anos: 21,0 (4,3)	5-10 anos: 7,3 (1,0)		5-10 anos: 7 (1,5)					
				>10 anos: 15,2 (2,3)	>10 anos: 52,3			>10 anos: 22,4 (4,2)	>10 anos: 7,7 (0,8)		>10 anos: 11,3 (2,3)					
Papadopoulou-Marketou et al.^24^	Comitê de Ética do Hospital Infantil Aghia Sophia	Grécia	Acompanhamento observacional	DM1: 13,1 (3,2)	DM1: 57,1	105	DM1 e Controles saudáveis	N/A	DM1: 8,36 (1,7)	Não	4,59 (3,49)	Sim	RAC	(TFGe)DM1: 90,72 (19,8)	Cistatina C sérica	ELISA, imunoensaio nefelométrico
				Controle: 12,8 (6,6)	Controle: 42,8				Controle: 4,7 (0,4)					Controle: N/A		
Papadopoulou-Marketou et al.^25^	Comitê de Ética do Hospital Infantil Aghia Sophia	Grécia	Acompanhamento prospectivo transversal de longo prazo	DM1: 13,9 (1,9)	DM1: 56,14	106	DM1 e Controle saudável	N/A	DM1: 9,1 (1,8)	Não	5,4 (3,3)	Sim	RAC	(TFGe) DM1: 110,1 (14,6)	Cistatina C sérica	ELISA, imunoensaio nefelométrico
				Controle: 10,5 (3,3)	Controle: 57,14				Controle: 4,65					(TFGe) Controle: 103,5 (14,5)		
Piani et al.^26^	N/A	EUA	Transversal	11,0 (4,0)	52,5	40	Pacientes com CAD	18,7 (4,5)	12,7 (1,9)	Sim	N/A	Não	N/A	(TFGe) 139,5 (5,7)	Cistatina C sérica	Imunoensaio tipo sanduíche
Salem et al.^27^	MS/15.12.52	Egito	Caso-controle	Normoalbuminúrico: 12,9 (1,9)	Normoalbuminúrico: 62,1	90	DM1 (normoalbuminúrico e microalbuminúrico) e Controle	N/A	Normoalbuminúrico: 7,4 (0,6)	Não	N/A	Sim	RAC	(creatinina) Normoalbuminúrico: 103,1 (33,3)	Cistatina C sérica	ELISA
				Microalbuminúrico: 13,4 (1,5)	Microalbuminúrico: 54,8				Microalbuminúrico: 7,9 (0,6)					(creatinina) Microalbuminúrico: 100,7 (27,9)		
				Controle: 13,8 (2,4)	Controle: 36,7				Controle: 4,9 (0,3)					(creatinina) Controle: 113,9 (30,2)		
Słomin´ski et al.^28^	N/A	Polônia	Coorte	Sem nefropatia: 12,7 (2,7)	50	240	Sem nefropatia e com nefropatia	Sem nefropatia: 18,9 (2,4)	Sem nefropatia: 8,4 (1,6)	Não	Sem nefropatia: 7,0 (2,8)	Sim	N/A	(TFGe) Sem nefropatia: 121 (38)	Cistatina C sérica	ELISA
				Nefropatia: 13,7 (3,6)				Nefropatia: 19,7 (2,8)	Nefropatia: 8,3 (1,2)		Nefropatia: 8,8 (3,0)			(TFGe) Nefropatia: 110 (28)		
Trutin et al.^29^	N/A	Croácia	Transversal	13,6 (3,8)	39	75	DM1	N/A	7,6 (1,2)	Não	6,3 (3,8)	Sim	N/A	(TFGe) 110 (19,2)	Cistatina C sérica	PETIA

Abreviaturas – RAC: Relação albumina/creatinina; IMC: índice de massa corporal; CAD: Cetoacidose Diabética; ELISA: Ensaio imunoenzimático; TFG: Taxa de Filtração Glomerular; HbA1C: Hemoglobina A1C; ID: identificação; N/A: Não Aplicável; PETIA: Imunoensaio turbidimétrico aprimorado por partículas; RPC: Relação proteína/creatinina; DP: diferença padrão; DM1: Diabetes Tipo 1; DM2: Diabetes Tipo 2; UK: Reino Unido; EUA: Estados Unidos da América.

Nota – TFG calculada com creatinina, cistatina C ou inulina ou TFGe calculada com creatinina (mL/min/1,73 m^2^).

### Avaliação da Qualidade dos Estudos Incluídos

A avaliação da qualidade dos 11 estudos incluídos utilizando a escala NOS é apresentada na Tabela 2. Apenas quatro estudos foram classificados como de alta qualidade^19,22,23,28^, cinco estudos como de qualidade moderada^20,21,24,25,27^ e dois foram considerados de baixa qualidade^26,29^.

**Tabela 2:** Avaliação de qualidade dos estudos incluídos

Primeiro autor & ano	A definição do caso é adequada?	Representatividade dos casos	Seleção de controles	Definição de controles	Comparabilidade de casos e controles com base no desenho ou na análise	Determinação da exposição	Mesmo método de determinação para casos e controles	Taxa de não-resposta	Escore total
Chae 2012^19^	*	*	*	*	*	*	*		7/9
Franchini 2015^20^	*	*		*	*	*	*		6/9
Maahs 2011^21^	*	*			*	*	*		5/9
Marcovecchio 2014^22^	*	*	*	*	*	*	*		7/9
Nilsson 2024^23^	*	*	*	*	*	*	*		7/9
Papadopoulou-Marketou 2014^24^	*	*		*	*	*	*		6/9
Papadopoulou-Marketou 2017^25^	*	*		*	*	*	*		6/9
Piani 2021^26^	*	*				*			3/9
Salem 2020^27^	*	*		*	*	*	*		6/9
Słomin´ski 2018^28^	*	*	*	*	*	*	*		7/9
Trutin 2022^29^	*	*				*			3/9

### Análise do Desfecho Primário

Um total de quatro estudos avaliou a associação entre os níveis séricos de cistatina C em indivíduos com DM1 e controles saudáveis. A estimativa agrupada indicou que o nível médio de cistatina C sérica no grupo DM1 foi 0,04 mg/L superior ao do grupo controle. Entretanto, essa diferença não foi estatisticamente significativa (Figura 2). Além disso, houve uma heterogeneidade substancial entre os estudos (I^2^ = 98%), sugerindo uma variabilidade significativa nos resultados. Não foi possível realizar uma análise da cistatina C urinária, uma vez que nenhum dos estudos incluídos a investigou.

**Figura 2:** Gráfico de floresta avaliando o papel prognóstico da cistatina C sérica na doença renal diabética (DRD).

Uma análise de sensibilidade (Figura 3) revelou que a omissão do estudo de Salem et al.^27^ reduziu substancialmente a heterogeneidade. Uma exploração mais detalhada utilizando o gráfico de Baujat (Figura 4) confirmou que tal estudo^27^ teve um impacto notável tanto no resultado geral quanto na heterogeneidade. Apesar de sua influência significativa, o estudo não foi excluído da meta-análise devido à sua importância na estimativa do efeito geral. A avaliação do viés de publicação não foi estatisticamente significativa (p = 0,1526).

**Figura 3:** Gráfico de floresta avaliando uma análise de sensibilidade do desfecho primário.

**Figura 4:** Gráfico de Baujat da heterogeneidade no desfecho primário.

### Análise de Desfechos Secundários

Pacientes com DM1 apresentaram níveis de PAD 3,95 mmHg superiores aos dos controles saudáveis, e essa diferença foi estatisticamente significativa (IC95% = [2,51–5,39], I^2^ = 39%) (Figura 5). Em contrapartida, a diferença média nos níveis de PAS entre os dois grupos não alcançou significância estatística (Figura S1). A RPC urinária, o IMC e os níveis de HbA1C, a duração do DM1 e o tipo de insulinoterapia aplicada em pacientes com DM1 não puderam ser examinados por não haver dados suficientes.

**Figura 5:** Gráfico de floresta avaliando os níveis de pressão arterial diastólica (PAD) em pacientes com diabetes mellitus tipo 1 (DM1) versus controles saudáveis.

## Discussão

A nefropatia causada pelo DM1, também conhecida como DRD, é uma complicação comum e bem descrita. Os pacientes pediátricos com DM1 devem ser avaliados aos 11 anos de idade com 2 a 5 anos de duração do DM1 ou na puberdade^30^. A manifestação mais precoce da DRD é a albuminúria, mas alguns pacientes podem apresentar um tipo específico de DRD normoproteinúrica [relação albumina/creatinina (RAC) urinária ≤30 mg/g e TFGe <60 mL/min/1,73m^2^]^31,32^. A DRD é geralmente detectável após o desenvolvimento de microalbuminúria ou pela redução da TFG em pacientes com DM1^3,33^. No entanto, a microalbumina na urina pode ser afetada pela retenção urinária, hipertensão arterial e infecções do trato urinário^34^. Além disso, a TFG é estimada principalmente pela creatinina sérica, que pode ser afetada pela massa muscular, sexo, idade e uso de medicamentos^35^. A TFGe calculada com base na creatinina é, com frequência, considerada estreitamente correlacionada com a TFGe real. No entanto, ela apresenta imprecisões, especialmente em crianças, devido aos fatores etnia, massa muscular, idade, sexo e diferenças na dieta^36^. A cistatina C atua como um marcador endógeno alternativo que permanece independente da massa muscular, da atividade física e da ingestão proteica, exibindo influência mínima por parte da idade e do sexo^36,37^. Considerando as alterações hormonais e de crescimento durante a adolescência, levantamos a hipótese de que a cistatina C possa ser um preditor mais eficaz da progressão da DRC do que a TFGe nessa faixa etária. Portanto, a aplicação de novos marcadores de DRD na prática clínica tornou-se necessária. A meta-análise de Liao et al.^35^ revelou que a cistatina C tem grande valor diagnóstico para a DRD em adultos com DM1 e DM2, já que apenas dois dos estudos incluídos avaliaram o papel prognóstico da cistatina C em pacientes pediátricos com DM1^19,27^.

Nossa revisão sistemática incluiu todos os estudos disponíveis que examinaram o papel da cistatina C em pacientes pediátricos com DM1. Nenhum outro estudo, consenso ou parecer de especialista revisou ou analisou esse tema, incluindo todos os estudos disponíveis de pacientes com DM1 com idade inferior a 21 anos, de acordo com as diretrizes *Bright Futures* da *American Academy of Pediatrics* ^38^.

Apenas cinco dos 11 estudos da revisão sistemática compararam diretamente pacientes com DM1 e um grupo controle saudável^20,21,24,25,27^. Os demais estudos compararam pacientes com DM1 a pacientes com DM2^19^ ou a outros pacientes com DM1 (com ou sem albuminúria^22^, de acordo com a duração do DM1^23^, com injúria renal aguda durante a cetoacidose diabética^26^, com ou sem DRD^28^, e com risco para DRD^29^).

Cinco estudos desta revisão constataram que a cistatina C sérica apresentou correlação positiva ou significância estatística com o prognóstico ou diagnóstico de comprometimento renal em pacientes pediátricos com DM1^19,23,24,27,29^. Especificamente, Chae et al.^19^ demonstraram que a cistatina C sérica esteve mais elevada em pacientes com DM e microalbuminúria (p = 0,04), embora não tenha havido separação entre pacientes com DM1 ou DM2 nessa comparação. No estudo de Nilsson e Dereke^23^, os níveis de cistatina C estiveram elevados em indivíduos com DM1 e duração da doença >5 anos (p < 0,001) entre os pacientes pediátricos com a condição. No estudo de Papadopoulou-Marketou et al.^24^, a cistatina C sérica apresentou correlação positiva com NGAL (p < 0,001) e creatinina (p = 0,009), mas correlação negativa com TFGe (p = 0,025) em pacientes com DM1 quando comparados a indivíduos saudáveis. Além disso, no estudo de Salem et al.^27^, os níveis de cistatina C sérica (p < 0,001) e TFGe estimada pela cistatina C (p < 0,001) foram significativamente mais elevados em crianças com microalbuminúria do que em crianças normoalbuminúricas com DM1 e controles saudáveis. Em crianças e adolescentes com DM1 no estudo de Trutin et al.^29^, a cistatina C foi significativamente associada ao risco estimado de DRD (p = 0,009). Em contraste, Franchini et al.^20^ mostraram que pacientes com DM1 apresentaram níveis mais baixos de cistatina C sérica em comparação ao grupo controle (p < 0,001), embora, após o ajuste para glicemia de jejum, a diferença entre os grupos tenha perdido significância. Além disso, nos estudos de Maahs et al.^21^ e Papadopoulou-Marketou et al.^25^, os níveis de cistatina C no soro foram semelhantes em pacientes com e sem DM1.

Apesar dos resultados significativos de estudos individuais sobre o benefício da cistatina C sérica na detecção ou no prognóstico da DRD em pacientes pediátricos com DM1, nossa revisão sistemática apresenta limitações que devem ser reconhecidas. Apenas quatro estudos foram incluídos na meta-análise^20,21,25,27^. Além disso, essa meta-análise não demonstrou significância estatística quanto ao papel da cistatina C sérica no prognóstico da DRD em pacientes pediátricos com DM1. Especificamente, apenas Salem et al.^27^ demonstraram que a cistatina C sérica pode levar à detecção precoce da DRD mesmo antes do desenvolvimento de albuminúria na infância. Uma possível explicação é que os estudos incluídos utilizaram abordagens diferentes para a avaliação da cistatina C, como TFGe com base na cistatina C, NGAL com base na cistatina C, creatinina com base na cistatina C e análise da curva ROC da cistatina C sérica, em vez de cistatina C sérica ou TFGe calculada com base na análise da cistatina C. A avaliação da qualidade do estudo incluído revelou que apenas quatro dos 11 estudos foram classificados como de alta qualidade^19,22,23,28^. Infelizmente, não foi possível realizar uma separação e análise adicional de pacientes com ou sem hiperfiltração e pacientes com ou sem microalbuminúria. Por fim, nenhum dos estudos incluídos mencionou a importância da cistatina C urinária como biomarcador tubular na DRD^10,11^ e a maioria dos desfechos secundários predefinidos não foi examinada.

## Conclusão

Em conclusão, embora estudos individuais tenham demonstrado algum benefício do uso da cistatina C sérica no prognóstico da DRD em pacientes pediátricos com DM1, a meta-análise dos estudos incluídos não alcançou significância estatística. Futuros estudos clínicos de grande porte, que examinem o papel prognóstico da cistatina C (tanto sérica quanto urinária), comparando crianças e adolescentes com DM1 e controles saudáveis, devem ser realizados para confirmar seu efeito benéfico no prognóstico da DRD.

## Data Availability

Os dados estão disponíveis online ou mediante solicitação ao autor correspondente.

## Material Suplementar

O seguinte material online está disponível para o presente artigo:

Tabela S1 – Estudos excluídos e motivo da exclusão.

Figura S1 – Gráfico de floresta avaliando os níveis de pressão arterial sistólica (PAS) em pacientes com diabetes mellitus tipo 1 (DM1) versus controles saudáveis.
